# Bats Are an Untapped System for Understanding Microbiome Evolution in Mammals

**DOI:** 10.1128/mSphere.00397-18

**Published:** 2018-09-19

**Authors:** Melissa R. Ingala, Nancy B. Simmons, Susan L. Perkins

**Affiliations:** a Richard Gilder Graduate School, American Museum of Natural History, New York, New York, USA; b Department of Mammalogy, Division of Vertebrate Zoology, American Museum of Natural History, New York, New York, USA; c Division of Invertebrate Zoology, American Museum of Natural History, New York, New York, USA; d Sackler Institute for Comparative Genomics, American Museum of Natural History, New York, New York, USA; University of Wisconsin—Madison

**Keywords:** Chiroptera, bats, macroevolution, microbial ecology, microbiota

## Abstract

Mammals evolved in a microbial world, and consequently, microbial symbionts have played a role in their evolution. An exciting new subdiscipline of metagenomics considers the ways in which microbes, particularly those found in the gut, have facilitated the ecological and phylogenetic radiation of mammals. However, the vast majority of such studies focus on domestic animals, laboratory models, or charismatic megafauna (e.g., pandas and chimpanzees). The result is a plethora of studies covering few taxa across the mammal tree of life, leaving broad patterns of microbiome function and evolution unclear. Wildlife microbiome research urgently needs a model system in which to test hypotheses about metagenomic involvement in host ecology and evolution. We propose that bats (Order: Chiroptera) represent a model system ideal for comparative microbiome research, affording opportunities to examine host phylogeny, diet, and other natural history characteristics in relation to the evolution of the gut microbiome.

## Abstract (precis)

Mammals evolved in a microbial world, and consequently, microbial symbionts have played a role in their evolution. An exciting new subdiscipline of metagenomics considers the ways in which microbes, particularly those found in the gut, have facilitated the ecological and phylogenetic radiation of mammals.

## PERSPECTIVE

Since the initiation of the Human Microbiome Project in 2008, there have been considerable advances in our understanding of microbial impacts on human health and disease (1). Similar studies in wildlife have not advanced at the same pace due to limited funding, despite the fact that researchers are increasingly recognizing critical links between humans and wildlife, both as key operatives in emerging infectious disease and as comparative models for human diseases. Studying animal microbiomes can also help us answer key questions about host evolution and ecology, but the vast majority of studies focus on a narrow range of host taxa. The result is that information about mammalian microbiome evolution derives from a depauperate set of snapshots that are neither evenly distributed across the spectrum of mammalian diversity nor representative of the array of different ecological niches found in mammals.

## MICROBIOME SAMPLING ACROSS THE MAMMAL TREE OF LIFE

Understanding how host-associated communities influence evolution—and evolve themselves—is an increasingly important goal for comparative biology and microbial ecology (2–4). The natural history of microbial gut communities in wild mammals is still poorly understood, but headway has been made in understanding patterns (5, 6), processes (7), and rates of divergence (8) in these systems over the last decade. Few studies sample host taxa in proportion to the percentage of mammal diversity that they comprise; for example, a large number of early microbiome studies focus on primates (9–13), which represent only ∼5% of the diversity of living mammals (14), but are our closest evolutionary relatives. Other charismatic megafauna are also overrepresented in the microbiome literature compared to their taxonomic diversity; the giant panda, a single species with a highly derived diet and lifestyle, has been the subject of at least five microbiome studies (15–19). Because it is not feasible to sample the microbiomes of all mammal species, it would be helpful to identify a clade that can be used as a tractable starting place for understanding patterns and processes of microbiome evolution across closely related species with different ecologies. With so many mammal groups to choose from, what would make any one clade an attractive choice? Here, we review the reasons why we believe bats are a good system within which to investigate new questions about the role of microbes in driving host evolution, physiology, and fitness.

## BATS ARE TAXONOMICALLY AND ECOLOGICALLY DIVERSE

**FIG 1:**
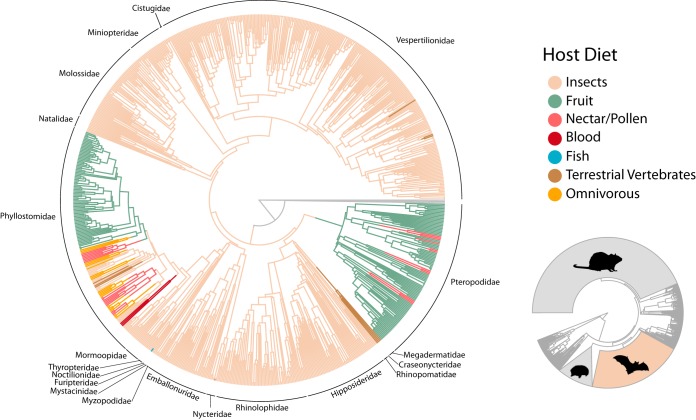
Host diet mapped onto the bat tree of life demonstrating multiple independent transitions to different diets (phylogeny from the work of Shi and Rabosky [62]). Bat families are labeled on the outer ring of the phylogeny. (Inset) Bat diversity relative to the rest of mammals. Approximately 70% of mammal diversity is contained in the orders Rodentia, Chiroptera (bats), and Eulipotyphla (hedgehogs, shrews, and allies). Mammal phylogeny from the work of Bininda-Emonds et al. (63).

Bats are unique among mammals as the only ones capable of powered flight. With nearly 1,400 described species, approximately 20% of all living mammal species are bats (20–22) (Fig. 1, inset). Bats also enjoy a cosmopolitan global distribution and are found on every continent save Antarctica (21). As such, they can serve as excellent models for understanding microbiome evolution at both local and landscape scales (23). Some evidence suggests that geographically widespread bat species show differences in microbiome structure among local populations (24), but the functional and fitness implications of this observation require more extensive analyses in taxonomic replicates. In addition to being widespread, bats also boast an astounding ecological diversity unrivaled by any other mammalian group (22, 25, 26). Diet is thought to be a major driver of microbiome structure and function (6), and few mammal groups compare to bats when it comes to dietary diversity. Within the order Chiroptera, different species have evolved to engage in frugivory, insectivory, carnivory, omnivory, nectarivory, and even hematophagy (27) (Fig. 1). In mammalian microbiome studies, there is often a strong correlation between host phylogeny and diet, making it difficult to parse apart the effects of diet and phylogeny on microbiome evolution (28). Bats provide a potential solution to this issue, because several feeding modes have evolved in parallel two or more times within the order. For example, frugivory evolved at least twice, once in the Pteropodidae and at least once in the Phyllostomidae, and similar instances of parallel evolutionary diet changes can be found for carnivory and nectarivory (Fig. 1). Importantly, many of these parallel transitions have occurred across the two major suborders of Chiroptera. Suborders Yinpterochiroptera and Yangochiroptera diverged over 60 million years ago, meaning that millions of years separate the evolution of frugivory in the phyllostomids (Yangochiroptera) and pteropodids (Yinpterochiroptera) (29). It is therefore important to test whether specialization on fruit proceeded via the same bacterial taxa or functions in these divergent host lineages. If not, alternative forces structuring microbiome communities in these bat groups, such as divergence in host physiology or behavior, may provide even more interesting explanations. Within each suborder, it would also be useful to assess microbiome structure as a function of dietary complexity among closely related frugivores. For example, do generalist species have functionally generalized microbiomes compared to those which feed on only a few plant species? Is there a standard toolkit of gut bacteria necessary for being frugivorous? More broadly, such an ecologically rich evolutionary system may prove useful in resolving the heated debate over whether host phylogeny or diet is more important in driving microbiome community structure and at what macroevolutionary scales each of these forces is most relevant (5, 6, 30).

## ECOSYSTEM-SCALE IMPLICATIONS FOR BAT MICROBIOMES

The ecological diversity of bats makes them incredibly economically important because they provide ecosystem services ranging from insect pest control (31, 32) to pollination of human fruit crops (33, 34). Understanding the microbiomes of bats can help shed light on their ability to carry out these and other vital ecosystem functions and can highlight potential dangers posed by rapid and continued habitat destruction. It has been shown in one species of primate that poor-quality habitats are associated with gut microbiomes that produce fewer short-chain fatty acids and have lower hydrogen metabolism than microbiomes of hosts living in higher-quality habitats (35); such studies in bats may likewise reveal troubling patterns of lower microbiome fitness in suboptimal habitats. Because nontrivial proportions of bat microbial community members are absent from conventional 16S databases (36), broad-scale studies also represent an opportunity for microbial taxonomists to discover, describe, and annotate the functions of novel bacterial species associated with bat hosts. With sufficient whole-genome coverage, it may be possible to discover even new candidate phyla of bacteria fulfilling important roles in host ecosystems. Using bats as a model clade, future microbiome studies could address critical questions including the following: do mammals with the same feeding habits contain the same taxonomic or functional consortia? Do single mammal species have the same consortia across their ranges, and if not, why do these differences arise? Do degraded habitats have a measurable impact on the fitness of their hosts via the microbiome, and can we detect wide-scale dysbiosis at the ecosystem level? Such studies will generate new questions and hypotheses about the myriad ways in which microbes shape life on earth.

## BAT MICROBES CAN SHED LIGHT ON DISEASE, IMMUNITY, AND LONGEVITY

Bats and their microbes are increasingly recognized as important components of zoonotic disease cycles (37, 38). A few studies have identified potentially pathogenic members of the excreted bat microbiome such as *Bartonella* spp. (39, 40) and *Leptospira* spp. (41). Bats are also known or suspected to be the reservoir of several viruses that are lethal to humans, such as severe acute respiratory syndrome (SARS), Ebola, and rabies viruses (42–44), as well as of *Plasmodium* parasites closely related to those in rodents that are used as models to study malaria (45). Genomic insights have generated plausible explanations for how bats may have evolved to harbor such deadly microbes (e.g., reference 46), but in spite of abundant evidence that the microbiome interfaces directly with the host immune system (47, 48), there has not yet been an integrative study addressing whether microbial symbionts contribute to bats’ innate ability to act as pathogen reservoirs. As an additional axis of variation, bats which have flexible roosting habits can be found in close proximity to humans and may potentially swap microbes with humans and their companion animals (49). Bats may transfer microbes to livestock when they exist in close proximity (e.g., pigs consuming partially eaten fruits dropped by fruit bats [50]) or use the same habitats (e.g., horses coming into contact with bat droppings in pastures [51]). Studying bat microbiomes would therefore have obvious public health implications and could help to explain the epidemiology of emerging infectious diseases.

Similar avenues of research can also consider what impact, if any, the host microbiome has on susceptibility of bats to white nose syndrome (WNS), a frequently fatal cutaneous infection that has reduced hibernating bat populations by up to 90% in North America (52). Because not all individuals are killed by the infection, there may be selection on the skin microbiomes of surviving individuals to become enriched with antifungal bacteria. Indeed, one study discovered that in WNS-positive populations, the skin microbiome of bats was enriched with *Rhodococcus* and *Pseudomonas* spp., which are known to have antifungal activity (53). Additional studies in this area can answer the questions of how exactly these bacteria inhibit the growth of the causative agent of WNS and what enrichment of the microbiome with these bacteria might mean for the long-term survival of affected host populations.

Bat microbiomes can be used more generally to understand the links between the microbiome and the evolution of other phenomena of interest, such as immunity and longevity. To date, studies addressing the link between host aging and the microbiome in humans and lab animals have uncovered direct links between microbial metabolic products and life span of the host (54). Bats represent an exciting system in which to test for links between the microbiome and aging because they are exceptionally long-lived for a mammal of their size (55, 56). Mice are conventional model mammals, but the commonly used BALB/c mouse strain has a life span of about a year and half, making studies of longevity in these animals rather short-lived (57). Bats of comparable mass can achieve life spans of up to 40 years, and many are philopatric to particular roosts, making repeat sampling of individuals throughout their lifetimes possible (58, 59). Because these animals’ microbiomes can be sampled nonlethally, they are inherently attractive for such studies (36). However, it is worth noting that these animals are especially sensitive to disturbance during hibernation, so experimental designs should minimize unintended disturbance of roosts, particularly in areas where white nose syndrome has decimated bat populations (60). It may also be possible to keep bats in captive colonies in order to sample them throughout successive years of their lives. Recent evidence suggests that metabolites produced by gut microbes in bats might offset the oxidative damages incurred during active flight, resulting in downstream impacts on aging (61). However, many questions still remain. By what mechanism does the microbiome help to extend life span, and is this pattern consistent across mammals? How does interindividual variation impact the relationships between longevity and microbiome community structure? We believe that studies of bat microbiomes can help to answer these important questions and more.

## FUTURE DIRECTIONS

Bats represent an untapped resource for understanding microbiome evolution in mammals. Because of their exceptional diversity, longevity, and ecological importance, we believe that studies of their microbial symbionts will reveal exciting new roles for microbes in driving host evolution and fitness and may help us to better understand the dynamics of emerging zoonotic pathogens. We provide applications of bat microbiome research in the hopes that more researchers will realize the potential that this system has to offer. Multi-omics approaches can be used to parse apart the contributions of host genome, metagenome, and microbial metabolites to the processes described above, and as the costs of these methods continue to decrease, such studies will only become more feasible. The results of studying bat microbiomes using these approaches will undeniably advance the fields of host-microbe interactions, comparative physiology, and public health.
